# ARD1/NAA10 acetylation in prostate cancer

**DOI:** 10.1038/s12276-018-0107-0

**Published:** 2018-07-27

**Authors:** Katherine J. Kuhns, Guanyi Zhang, Zehua Wang, Wanguo Liu

**Affiliations:** Department of Genetics, Stanley S. Scott Cancer Center, Louisiana State University Health Sciences Center, New Orleans, LA 70112 USA

**Keywords:** Protein folding, Protein folding

## Abstract

Prostate cancer (PCa) is the second most common cancer in men. Androgen receptor (AR) signaling pathway plays a crucial role in prostate development and homeostasis. Dysregulation of this pathway activates AR leading to PCa pathogenesis and progression. AR binds testosterone and other male hormones, which then undergoes post-translational modification for AR nuclear translocation and transcriptional activation. AR activation by post-translational modification is thus imperative for PCa cell growth and survival. Identification and understanding of the pathological and mechanistic roles of AR modifications may increase our understanding of AR activation in PCa and provide new therapeutic options. Recently, AR acetylation has been described as an important step for AR activation. Upregulation of several acetyltransferases has been reported to be associated with PCa progression. Herein, we provide a general understanding of AR acetylation, with a special emphasis on ARD1, and potential therapies that may be exploited against the ARD1–AR axis for PCa treatment.

## Prostate cancer: Preventing androgen receptor activation

Blocking the addition of an acetyl group to androgen receptors by Arrest-defect-1 protein (ARD1) might be an effective strategy for halting prostate cancer progression. High levels of ARD1 are found in many types of cancer and previous studies have shown that it contributes to prostate cancer (PCa) cell proliferation and survival by stimulating androgen receptor activity. Wanguo Liu and colleagues at Louisiana State University Health Sciences Center, New Orleans, USA, review current knowledge of the regulation and effects of ARD1 on tumor formation. The ARD1-mediated post-translational modification of androgen receptors causes them to move from the cytoplasm to the nucleus where they activate the expression of genes involved in tumor growth. Compounds that inhibit this modification could offer a new treatment option for patients with prostate cancer.

## Introduction

Arrest-defect-1 protein, ARD1, also known as Naa10, is the catalytic subunit of *N*-acetyltransferase (NAT) group A (NatA)^1^. The complex mainly functions as an N-terminal acetyltransferase to transfer an acetyl group from acetyl-coenzyme A to the α-amino group of serine, glycine, alanine, threonine, valine, and cysteine side chains at the N-terminus of proteins^2–4^. Following acetylation of the α-amino group, the initiator methionine is removed via methionine aminopeptidases^2–4^. In addition, the complex also possesses lysine acetylation activity to catalyze acetylation of ε-lysine on internal residues of proteins^1,5,6^. By acetylating specific substrate proteins, ARD1 plays critical roles in cell-cycle arrest, apoptosis, hypoxia, autophagy, and cell proliferation^6–12^. Although one report indicated that ARD1 is unable to acetylate lysine within a protein, the acetylation of ε-lysine by ARD1 has been demonstrated to occur in many proteins such as Hif1, Hsp70, Hsp90, and AR by in vitro and in vivo analytical approaches (Table 1)^6,9,11,13–22^. Moreover, a recent report further supports the capability of ARD1 to acetylate ε-lysine by demonstrating a critical role of Hsp70 acetylated by ARD1 at lysine 77 in response to stress^18^. It is thus possible that ε-lysine acetylation by ARD1 and its effects on dysregulating key cellular signaling are far more common in proteins than previously thought.

**Table 1:** ARD1-mediated *ε*-amino acid acetylation in proteins

	Protein	*ε*-Amino acid	Function	Discovery method	Citation
1	AR	K618	Cell proliferation & tumorigenesis	In vivo & in vitro acetylation assays	DePaolo et al. (2016)^13^
2	PGK1	K388	Tumor formation	MS	Qian et al. (2017)^14,15^
3	HIF-1α	K532	Cell proliferation	In vitro acetyltransferase assay	Jeong et al. (2002)^9^
4	Aurora Kinase A	K75 & K125	Cell proliferation & migration	In vitro acetylation assay	Vo et al. (2018)^16^
5	SAMHD1	K405	Cell proliferation	In vitro acetylation assay & LC-MS/MS	Lee et al. (2017)^17^
6	Hsp70	K77	Protein homeostasis	In vitro acetylation assay & LC-MS/MS	Seo et al. (2016)^18^
7	MSRA^a^	K49	Oxidative stress	In vitro acetylation assay & LC-MS/MS	Shin et al. (2014)^6^
8	Runx2^a^	K225	Osteogenesis	LC/MS & antibody	Yoon et al. (2014)^19^
9	MLCK^a^	K608	Tumor migration and metastasis	Site directed mutagenesis & in vitro acetylation assay	Shin et al. (2009)^20^

^a^Magin et al.^21^ found these proteins to be chemically, not enzymatically, acetylated

## ARD1 in human cancers

ARD1 is upregulated in many different types of cancers such as lung, colon, liver, bladder, and PCa^12,23–26^. Overexpression of ARD1 correlates with aggressiveness of tumors^24^. Ectopic expression of ARD1 increases, while silencing ARD1 significantly reduces cell proliferation, providing strong evidence for the role of ARD1 as an oncoprotein in cancer^5,12^. The oncogenic properties of ARD1 are exerted through the regulation of Cyclin D1 in lung cancer, acetylation of β-catenin in colorectal cancer, and recruitment of DNMT1 to the E-cadherin promoter in which DNMT1 silences the transcription of the tumor suppressor E-cadherin^11,27^. To date, only one report has shown that ARD1 could reduce cell growth and induce autophagy in a subgroup of breast cancer patients by inhibiting mTOR signaling^10^. The role of ARD1 in tumorigenesis is thus dependent on its direct target proteins in different tumor types and tissues.

## Pathological role of ARD1 in PCa

### AR acetylation in PCa

Along with common post-translational modifications such as phosphorylation and methylation seen in many proteins, AR can also be acetylated^28^. Many investigators have demonstrated that AR acetylation enhances AR activity and PCa cell survival^13,29^. Following acetylation, nuclear ligand-bound AR at the androgen response element (ARE) undergoes architectural changes via chromatin remodeling into an open conformation^12,29,30^. The open conformation induces recruitment of RNA Pol II and other coregulators to the AR complex^12,29,30^. The open conformation of the complex induced by acetylation results in increased AR-mediated transcription of AR target genes necessary for PCa progression^12,29–31^. Several acetyltransferases that are known to modulate AR activity include, but are not limited to, p300, p300-CBP associated factor (P/CAF), Tat interactive protein 60 (TIP60), and ARD1^12,13,29,32–35^.

Work by the Pestell group^32^ first elucidated that ligand-induced AR function was enhanced by p300 and P/CAF because of their intrinsic histone acetyltransferase (HAT) function. Specifically, p300 acetylated AR at the conserved lysine motif ^630^KLKK^633^ in the hinge region of AR that mimics a conserved acetylation motif RXKK across multiple species^12,31,32^. Moreover, the location of the acetylation motif in AR is seen in other non-histone proteins that serve as HAT substrates^32^. The individual lysine residues, 630, 632, and 633 are not only acetylated by p300, but they have also been found to be acetylated by other acetyltransferases such as P/CAF and TIP60^4,12,32,33^.

Identification of an in vivo or pharmacological inhibitor that inhibits p300 acetylation of AR may offer new insight into the development of therapies designed to inhibit AR acetylation by p300. Intriguingly, the Pestell group^36^ demonstrated that p300 is repressed by the deacetylase SIRT1. Finding that SIRT1 and p300 directly interacted with one another led to the observation that SIRT1 represses p300’s transactivation activity via lysine 1020 (K1020) and lysine 1024 (K1024) in the cell cycle regulatory domain 1 (CRD1)^36^. Later they were able to demonstrate that SIRT1 can inhibit AR-dependent gene expression by modulating AR acetylation at a p300 acetylation site, lysine 630 (K630)^37^. Additionally, a late-breaking paper from the Denu group^38^ identified another sirtuin, SIRT2, as having reduced expression in castration resistant prostate cancer (CRPC). Decreased expression of SIRT2 contributes to increased p300 activity and hyperacetylation of histone 3-lysine 18 (H3K18), an epigenetic modification unique to CRPC^38^. Further studies elucidating the interaction between p300 and sirtuins will be significant for understanding if inhibiting AR activation as a result of acetylation thereby prevents PCa development and quite possibly, progression to CRPC.

### ARD1 acetylates AR for AR activation

We previously reported that ARD1 is upregulated in all PCa cell lines and in 97% of evaluated primary PCa tumor tissues^12^. Like p300, the expression level of ARD1 seems to correlate with tumor grades of PCa (unpublished data)^12^. Moreover, the expression level of ARD1 is higher in PCa cells with AR than those without AR. Further experiments also demonstrated that ARD1 levels can be induced by synthetic androgen (R1881) in a dose-dependent manner in LNCaP cells, but not in AR-null PC-3 and DU-145 cells, suggesting that upregulation of ARD1 in PCa is induced by androgen in an AR-dependent manner^12^. However, how androgen induces ARD1 protein level or ARD1 stability in PCa remains to be elucidated.

**Fig. 1:**
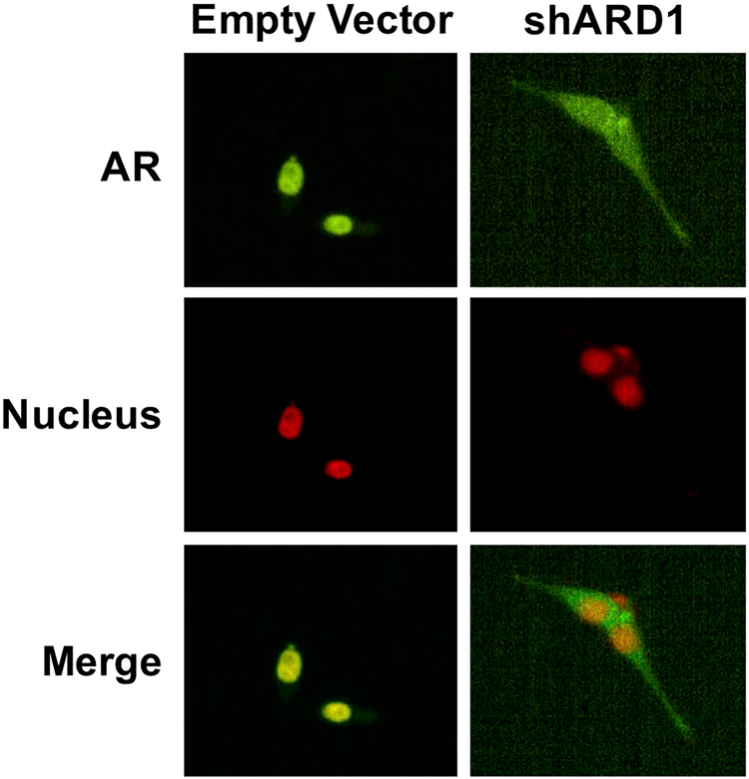
ARD1 knockdown in LNCaP inhibits endogenous AR nuclear translocation. Nuclear translocation of AR proteins in LNCaP cells was analyzed by confocal fluorescence microscopy. The AR antibody (Santa Cruz Cat# SC-816) and the Alexa Fluor 488 secondary anti-rabbit IgG antibody (Cell Signaling Cat# 4412) were used to probe AR in PCa cells. LNCaP cells were cultured in RPMI-1640 medium with 10% FBS before knockdown. Lentivirus for ARD1 knockdown was generated using the Lipofectamine 3000 (ThermoFisher Scientific) system. The empty vector and knockdown cells were cultured in phenol red-free RPMI-1640 media supplemented with 10% charcoal stripped-FBS and were fixed with 4% paraformaldehyde. Nuclei were stained with 2.5 μM DRAQ5 (Cell Signaling Cat# 4084). *N* = 3, representative images shown. Confocal images were obtained using an Olympus Confocal Laser Microscope with a ×60 oil-immersion objective on a Z-stage

The pathological roles of the upregulation of ARD1 in PCa were demonstrated via experiments overexpressing or silencing ARD1. We showed that ARD1 is required for AR target gene transactivation, PCa cell proliferation, and xenograft tumor growth^12^. Mechanistically, ARD1 forms a complex with AR and Hsp90 leading to AR acetylation at lysine 618 (K618) by ARD1 resulting in AR-Hsp90 dissociation, AR nuclear translocation, AR target gene expression, and androgen-dependent PCa tumorigenesis^13^. Thus, ARD1 is an oncoprotein in PCa. Unfortunately, the previously published data only demonstrated that ARD1 activates AR in vitro. Here we provide in vivo evidence to show that knockdown of ARD1 in LNCaP cells inhibits nuclear translocation of endogenous AR (Fig. 1). However, as we observed in in vitro experiments, silencing of ARD1 in LNCaP cells only inhibits AR nuclear translocation in less than 40% of the cells. It is possible that silencing of ARD1 impairs other ARD1 acetylated proteins that regulate AR shuttling between the nucleus and cytoplasm. Identification of these ARD1 targets could enhance future therapeutic applications targeting the ARD1–AR axis in PCa.

Unlike p300 and TIP60, ARD1 does not acetylate the ^630^KLKK^633^ motif, instead, it preferentially acetylates the DNA binding domain (DBD) of AR^13^. The DBD of AR is comprised of 3-lysine-containing motifs^13^. Previous work from our lab demonstrated that K618 is the ARD1 acetylation target both, in vitro and in vivo, through site-directed mutagenesis via the generation of arginine substitutes^13^. As expected, mutations in both the K618 and ^630^KLKK^633^ acetylation sites abrogated almost all of AR acetylation^13^. However, it is intriguing that mutations in ^630^KLKK^633^ sites do not reduce AR acetylation while individual loss of the K618 acetylation site reduced AR acetylation level more than 80% as compared to WT-AR^13^. Because silencing of p300 does not reduce AR acetylation by ARD1, these data suggest that acetylation of K618 could be a necessary priming event for AR acetylation by p300.

We showed that ARD1-AR-Hsp90 form a complex in the cytoplasm^13^. However, the role of Hsp90 in the process of AR acetylation by ARD1 remains unknown. The Varshavsky group^22^ recently demonstrated that ARD1 yeast mutants have increased Arg/N-end rule pathway due to the absence of NatA Nt-acetylase. Consequently, this resulted in dysregulation of Hsp90 and its targets due to loss of N-terminal acetylation^22^. Moreover, it has been found that there is increased Hsp90 acetylation by ARD1 through its binding to Hsp90 under hypoxic conditions^39^. Combining these findings into the mechanism of ARD1-mediated AR activation, it is possible that hypoxic conditions observed in PCa may promote ARD1-Hsp90-AR trimer formation and enhance AR-Hsp90 dissociation via Hsp90 acetylation by ARD1. Although these hypotheses have not yet been tested in vivo in PCa cells, this putative mechanism may add another layer to our understanding of ARD1 in prostate tumorigenesis.

### Upstream and downstream regulators of ARD1 in PCa

**Fig. 2:**
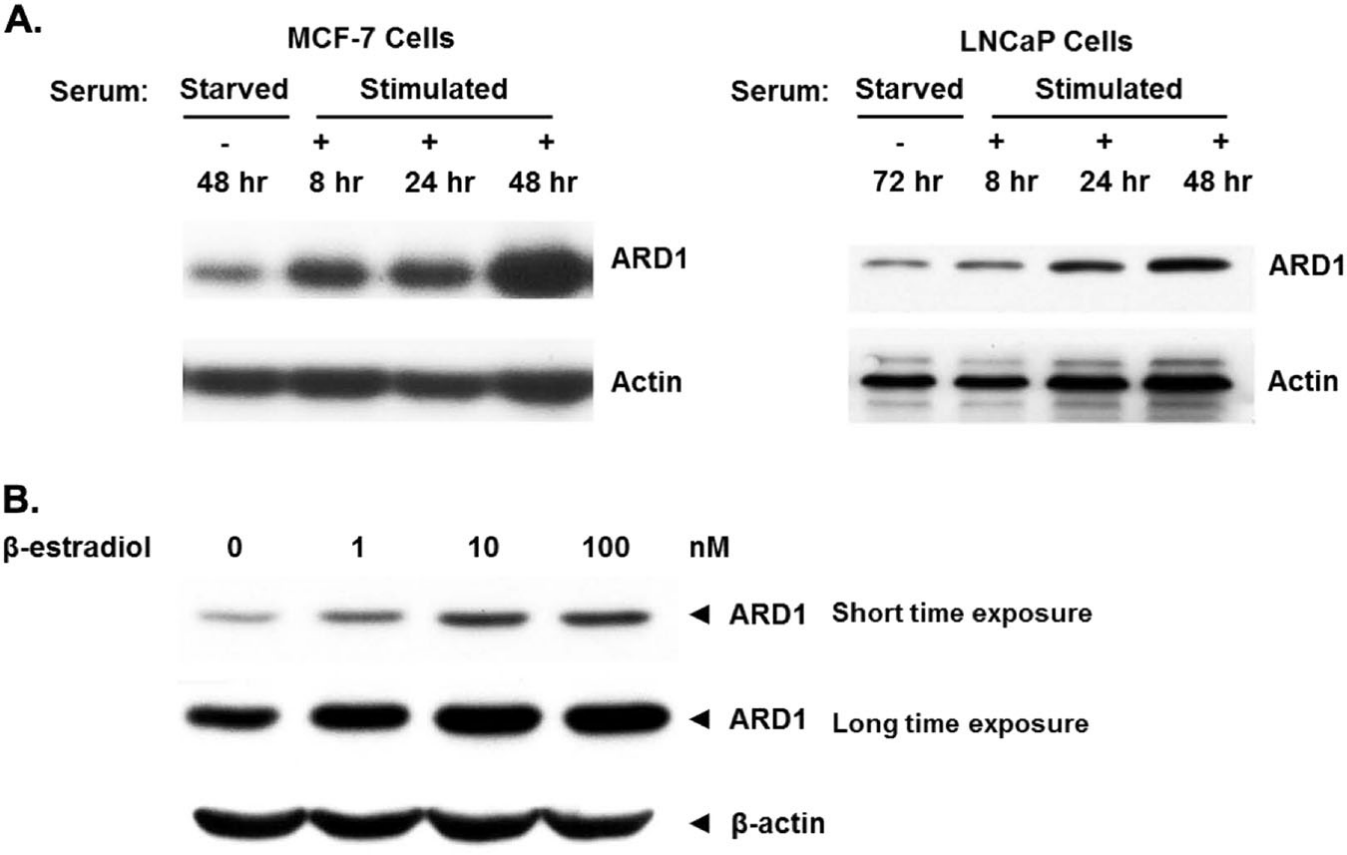
Hormone and growth factor upregulation of ARD1 in cancer. **a** MCF-7 and LNCaP cells were serum starved for 48 and 72 h, respectively, and subsequently re-stimulated with 10% FBS for 48 h. ARD1 expression increased with time exposed to serum treatment. **b** MCF-7 cells were treated with increasing concentrations of β-estradiol (estrogen) for short and long time exposures. ARD1 expression increased with increasing both treatment duration and concentration of estrogen. *N* = 3, representative blots shown. Whole-cell lysates were collected and run on gradient 4–20% SDS gels and bands were detected via the ECL method

Because ARD1 is upregulated in most cancers, understanding how regulators of ARD1 effect ARD1 expression and its targets will contribute greatly to further contextualizing ARD1’s role in cancer. Specifically, identification of ARD1 regulators in PCa will facilitate development of better chemotherapy strategies that repress ARD1 expression. ARD1 splice variants have intrinsic auto-acetylation function that is essential for its activation^40,41^. Auto-acetylation not only regulates enzymatic function in ARD1 variants, it also regulates cellular functions such as increased cellular proliferation as well as Cyclin D1 and β-catenin activation subsequently leading to tumorigenesis^40,41^. While the effects of auto-acetylation on cancer cell growth are irrefutable, auto-acetylation is not an encompassing contributor to the elevated ARD1 expression observed in tumors. Cell stimuli also contribute to elevated ARD1 expression. Besides androgen, serum can also induce ARD1 expression in PCa while estrogen induces ARD1 in breast cancer cells (Fig. 2). This suggests that ARD1 upregulation in cancer cells can likely be induced by hormones or growth factors. Reports have also indicated that elevated insulin-like growth factor 1 (IGF1) levels increase risk for PCa along with enhanced AR transactivation under androgen deprivation therapy (ADT) in which circulating androgen levels are very low^42^. It would be interesting to follow-up the serum data and determine if IGF1 and other well studied growth factors in PCa increase ARD1 expression. Moreover, this follow-up study could be extrapolated to determine if hormones and growth factors contribute to increased risk of CRPC following ADT.

**Fig. 3:**
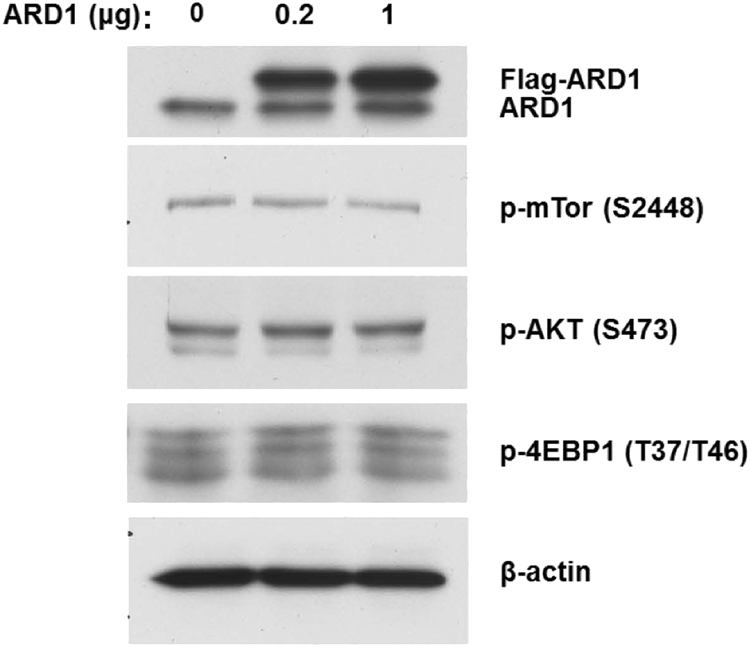
ARD1 does not inhibit mTOR signaling in prostate cancer. Increasing amounts of FLAG-ARD1 were transfected in PC-3 cells. ARD1 overexpression did not increase p-mTOR, p-AKT, and p-4EBP1 expression relative to endogenous ARD1 expression and its effect on downstream targets. Expression of the downstream mTOR target, p-4EBP1, under the mock ARD1 transfection condition, suggests ARD1 expression activates mTOR signaling in PCa as opposed to inhibition reported in breast cancer. *N* = 3, representative blots shown. The Lipofectamine 3000 (ThermoFisher Scientific) system was used for transfection. Whole-cell lysates were collected and run on gradient 4–20% SDS gels and bands were detected via the ECL method

Other reported regulators of ARD1 include IkB kinase (IKKβ) and tuberous sclerosis 2 (TSC2)^10,43,44^. IKKβ acts as an upstream regulator of ARD1 in which ARD1 physically interacts with IKKβ and serves as a substrate for IKKβ^43^. ARD1 is subsequently destabilized by IKKβ upon phosphorylation at serine 209 (S209) leading to proteasomal degradation^43,44^. The destabilization of ARD1 hence decreased the control of ARD1 on suppressing growth^43,44^. Conversely, TSC2 was identified as a downstream regulator of ARD1 in which ARD1 and TSC2 directly interacted with one another^10,43^. ARD1 acetylates TSC2 yielding subsequent stabilization^10,43^. Consequently, acetylated-TSC2 inhibited downstream mTOR signaling, thereby reducing cell proliferation and inhibiting tumorigenesis^10,43^. Although it has been demonstrated that ARD1 inhibits mTOR signaling in breast cancer, we have evidence that suggests that ARD1 activates mTOR signaling in PCa cells (Fig. 3). Despite the dichotomy between the effects of ARD1 on the mTOR pathway, we believe these differences occur because of the different tissues from which these cancers arise.

Vo et al.^16^ have recently reported that aurora kinase A (AuA) as another downstream target of ARD1. ARD1-mediated acetylation of AuA at lysines 75 and 125 promoted cell migration and cell proliferation via activation of the p38/AKT/MMP-2 pathways and cyclin E/CDK2 and cyclin B1, respectively^16^. Once again, the work elucidating AuA as a downstream target of ARD1 was demonstrated in breast cancer cells, MCF-7^16^. While this has not been investigated in PCa, it is likely there may be an effect observed because of the overlap between the nuclear hormone receptor-driven activation of both breast and prostate cancers.

Finally, Cdc25A, a phosphatase responsible for de-phosphorylating cyclin-Cdk complexes in the cell cycle, has been shown to interact with ARD1 and be acetylated by it biochemically and in vitro^45^. Both Cdc25A and ARD1 are known to be dysregulated in cancer^45^. While the mechanism describing the interaction between Cdc25A and ARD1 was recently reported for the first time, it has yet to be extrapolated and studied in in vivo cancer cell lines^45^. Collectively, the aforementioned regulators of ARD1 have convincing effects on tumorigenesis. Modulation of upstream regulators and growth factors of ARD1 may confer more control on increased ARD1 expression observed in PCa. Thereby repressing ARD1 by decreasing hormones and growth factors may disrupt interactions of ARD1 with downstream targets henceforth exerting more control over PCa tumorigenesis.

### Functional significance of ARD1 in nuclei

**Fig. 4:**
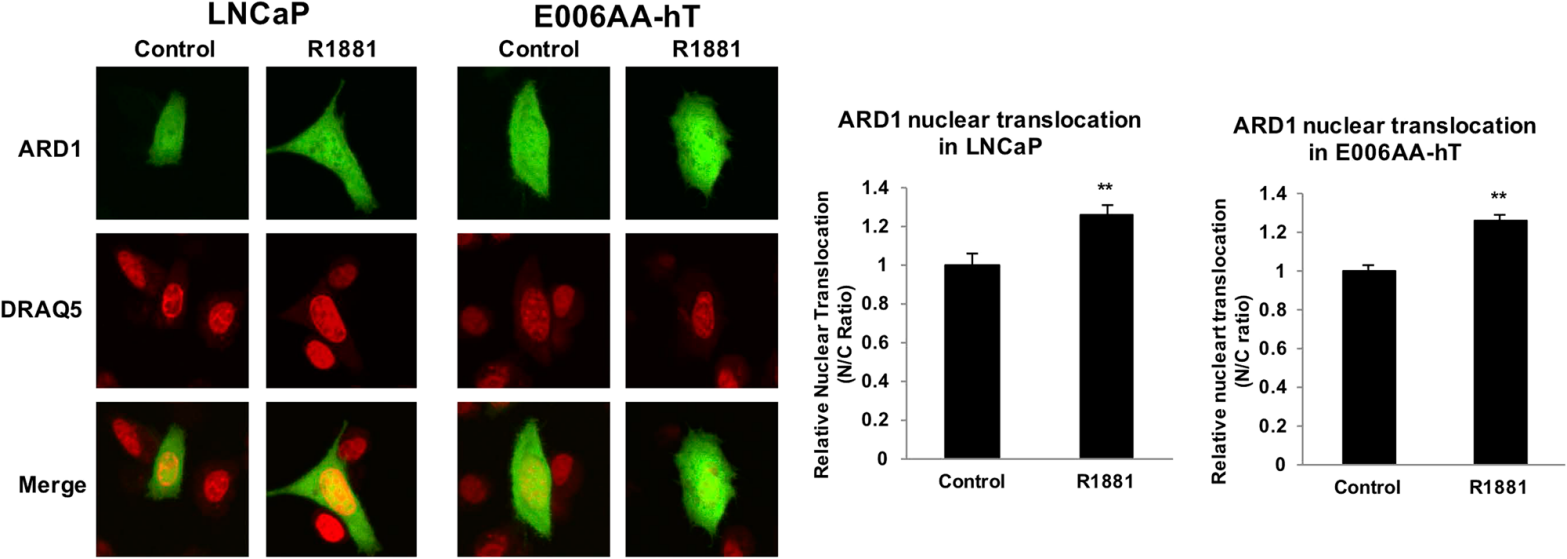
ARD1 nuclear localization in LNCaP and E006AA-hT cells. ARD1 nuclear localization is seen in LNCaP and E006AA-hT cells and increases with androgen treatment. Subcellular distribution of ARD1 proteins was analyzed by confocal fluorescence microscopy. The ARD1 antibody (Santa Cruz Cat# SC-3320) and the Alexa Fluor 488 Anti-rabbit IgG antibody (Cell Signaling Cat# 4412) were used to probe ARD1 in PCa cells. LNCaP and E006AA-hT cells were respectively cultured in phenol red-free RPMI-1640 and phenol red-free DMEM media supplemented with 10% charcoal-stripped FBS. The cells were treated with or without 1 nM R1881 for 24 h and subsequently fixed with 4% paraformaldehyde. The nuclei were stained with 2.5 μM DRAQ5 (Cell Signaling Cat# 4084). *N* = 3, representative images shown. Confocal images were obtained using an Olympus Confocal Laser Microscope with a ×60 oil-immersion objective on a Z-stage. An average of six fields with 10 cells per field were captured for each group. The NIH Image J Software was used to quantify the nuclear (N) and cytoplasmic (C) fluorescent signals. The ratios of nuclear to cytoplasmic (N/C) signals were calculated to demonstrate ARD1 protein nuclear translocation

The cellular distribution of ARD1 in cancer varies. As shown by Kuo et al.^43^, in certain breast cancer cells, ARD1 exclusively resides in nuclei. We measured the nuclear level of ARD1 in PCa cells and showed that about 50% of ARD1 is in the nucleus of LNCaP and E006AA-hT cells (Fig. 4). The level of ARD1 in the nucleus is even higher after exposure to androgen (R1881), suggesting that ARD1 probably translocates into the nucleus with acetylated AR. However, this needs to be further examined in an in vivo system. To date, a functional role for ARD1 in the nucleus is largely unknown. However, our data suggest that ARD1 may have significant nuclear function because we observe an equal distribution of ARD1 in both the cytoplasm and nucleus in approximately 50% of PCa cells. While most ARD1 targets are in the cytoplasm, work from Rual et al.^46^ provided us our earliest evidence of nuclear ARD1 with the protein–protein interaction network demonstrating that euchromatic histone-lysine-*N*-methyltransferase 2 (EHMT2), also referred to as G9a, interacts with ARD1. EHMT2 catalyzes the methylation of H3K9. It is possible that ARD1 may epigenetically modify histones to alter the transcriptionally active or repressive status of stress response genes in tumors to generate an environment supportive of tumor growth and invasion. Furthermore, we find that ARD1 interacts with proliferating cell nuclear antigen (PCNA) in the nucleus through mass spectrometry analysis of proteins immunoprecipitated with ARD1 in LNCaP cells (unpublished data). The interaction between ARD1 and PCNA may also be necessary to support tumor cell survival because PCNA is a critical scaffold protein that recruits other proteins that promote genomic integrity for DNA replication and repair. Although the functional role of ARD1 in the nucleus remains elusive, there is compelling evidence that ARD1 can indeed be found in the nucleus to interact with nuclear proteins for regulatory functions of cell signaling in cancer.

## ARD1 axis as a potential cancer therapeutic target

Based on the discovery that ARD1 serves as a novel AR acetyltransferase and regulates AR activity and PCa tumorigenesis, we postulate that first-line therapies should be designed to exploit the ARD1–AR axis. These potential therapies could prove to be more effective than the deleterious effects seen in canonical ADT in which some cases develop into CRPC after becoming refractory to ADT treatment. Because ADT inhibits androgen production and AR activation, it consequently inhibits beneficial functions of the AR that are needed in normal prostate cells and tissues. Development of a small-molecule inhibitor designed to inhibit the ARD1–AR interaction would prevent AR acetylation and AR activation. Furthermore, a treatment of this nature would also maintain ARD1-mediated acetylation at distant sites without perturbing signaling pathways dependent on protein acetylation from ARD1. Current acetyltransferase inhibitor therapy in cancer is via use of histone deacetylases (HDAC) inhibitors. The marginal efficacy of HDAC inhibitor therapy implores the need for a specific therapy to inhibit the ARD1–AR interaction via inhibition of AR acetylation.

Lastly, a second-line therapy designed to inhibit the AR–ARD1–Hsp90 complex dissociation as a result of ARD1 acetylation would offer another approach to treat PCa. Once again, we propose the development of another small-molecule inhibitor that would stabilize the trimer in the cytoplasm. Similar to HDAC inhibitor therapy in current use, Hsp90 inhibitor therapy is minimally effective. The advantages of this small-molecule therapy would prevent disruption of the chaperone functions of Hsp90 on its client proteins that help maintain normal cell homeostasis. Whereas both small-molecule inhibitor therapies we are proposing are designed to specifically inhibit different interactions along the AR–ARD1 axis, both therapies are ultimately intended to inhibit nuclear translocation of K618-acetylated AR to prevent target gene expression of genes necessary for androgen-dependent tumorigenesis.

## Conclusions and future perspectives

**Fig. 5:**
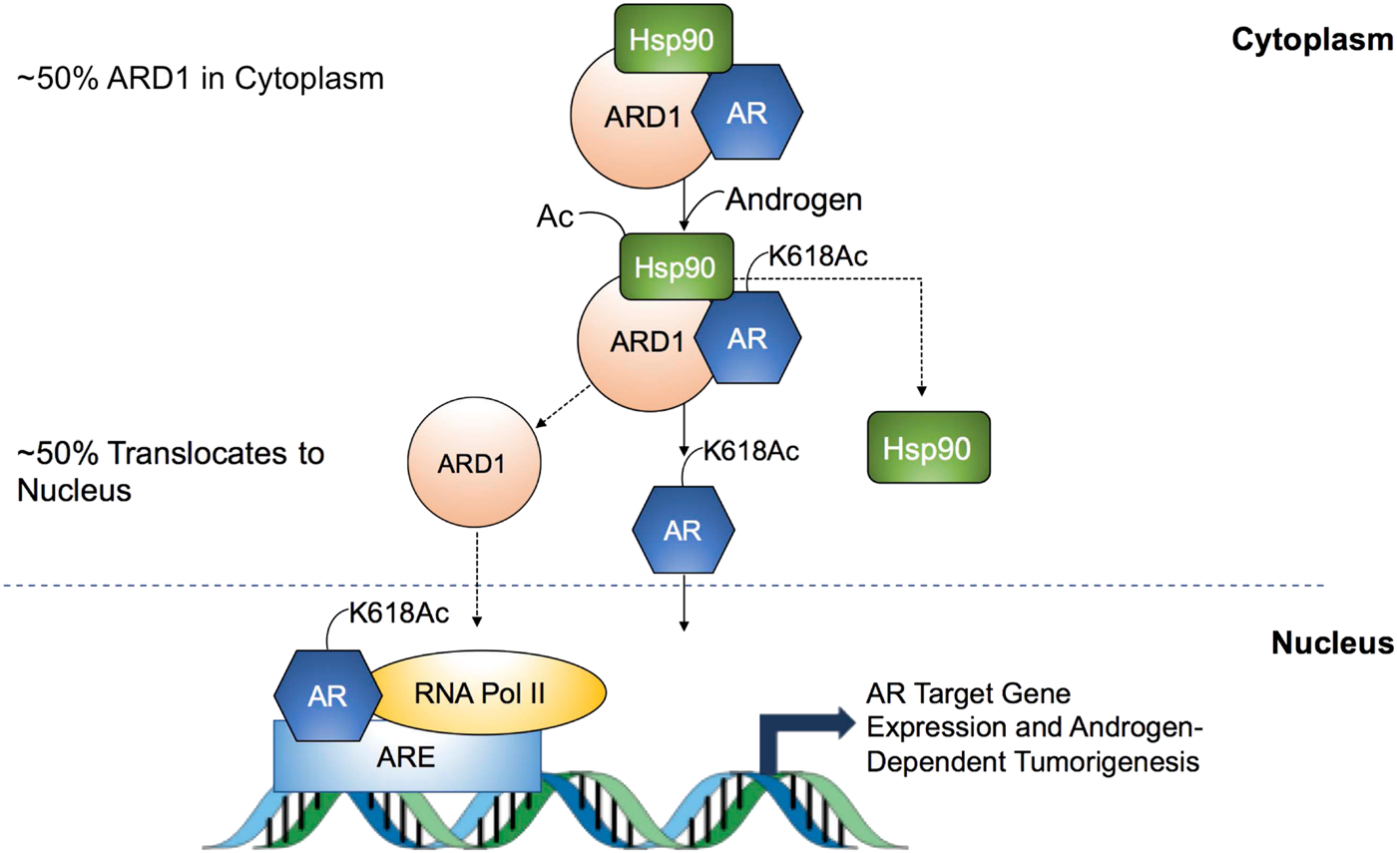
ARD1 acetylates AR for nuclear translocation and transactivation. AR, ARD1, and Hsp90 are complexed in the cytoplasm as a trimer. Upon androgenic stimulation, ARD1 acetylates Hsp90 and AR at K618, which results in dissociation of the complex. Hsp90 remains in the nucleus and acetylated AR translocates to the nucleus and binds to the Androgen Response Element (ARE) and RNA Pol II for transcription of AR target genes necessary for androgen-dependent prostate cancer tumorigenesis. While a majority of ARD1 resides in the cytoplasm, approximately 50% of ARD1 translocates to the nucleus with even more translocating after androgen exposure

In summary, ARD1 is an acetyltransferase that post-translationally modifies many proteins by acetylating alpha- or epsilon-amino acids. These modifications subsequently regulate many important cellular processes in cells that become deregulated and promote tumorigenesis in both a target- and tissue-specific fashion. In PCa, ARD1 acetylates AR at K618 to activate AR leading to AR nuclear translocation and prostate tumorigenesis (Fig. 5). The discovery of ARD1 molecular and pharmacological inhibitors that are tumor-specific will be essential to abrogating tumor growth while not inhibiting critical modifications by ARD1 at distal sites. Furthering our understanding of the mechanism by which ARD1-mediated AR acetylation results in oncogenic transformation in prostate cells may also yield significant insight into current treatment challenges for PCa and other cancers.

Several questions remain about ARD1 and its role in PCa tumorigenesis. First, growth factors play an important role in PCa progression^47–49^. Given the evidence that ARD1 can be upregulated by serum, identification of the growth factors that modulate ARD1 expression will be essential for lowering ARD1 levels in PCa. Furthermore, decreasing ARD1 expression through this method will also be beneficial for preventing progression to CRPC. Because elevated hormones during ADT treatment increase risk for CRPC, understanding the dynamics of growth factors and hormones on ARD1 expression may also enable us to ascertain the cellular conditions of the tumor environment that lead to CRPC. Secondly, AR splice variants, such as AR-V7, appear critical for CRPC progression and drug resistance. Because AR-V7 lacks p300 acetylation sites but retains the ARD1 acetylation site, it will be imperative to determine if ARD1 can acetylate AR splice variants. Moreover, it is also important to determine if the ARD1 acetylation event associates with CRPC progression and drug resistance. The determination of the interaction domain between ARD1 and AR splice variants will be crucial in the construction of a specific small peptide or identification of a small molecule to inhibit the possible acetylation event. Therefore, the potential roles of ARD1 in regulation of AR splice variants in CRPC are of great importance that requires further investigation. Finally, the roles of ARD1 in the nucleus remain largely unknown. We and others have shown that ARD1 is present in nuclei and interacts with nuclear proteins. The evidence that ARD1 interacts with G9a suggests that ARD1 may regulate histone function, supporting the idea that ARD1 could be an AR coactivator. Therefore, further elucidation of the roles and mechanisms of ARD1 on G9a and the histone complex is needed to yield new insight of ARD1 function in nuclei in PCa progression and drug resistance.
